# Substance use and other factors associated with COVID-19 vaccine uptake among people at risk for or living with HIV: Findings from the C3PNO consortium

**DOI:** 10.1016/j.pmedr.2023.102300

**Published:** 2023-06-24

**Authors:** Marjan Javanbakht, Lamia Khan, Brian Mustanski, Steve Shoptaw, Marianna K. Baum, Shruti H. Mehta, Gregory D. Kirk, Shenghan Lai, Richard Moore, M-J Milloy, Michele Kipke, Kanna Hayashi, Kora DeBeck, Suzanne Siminski, Lisa M White, Pamina Gorbach

**Affiliations:** a Department of Epidemiology, Fielding School of Public Health, University of California, Los Angeles, Los Angeles, CA, USA; b Institute for Sexual and Gender Minority Health and Wellbeing and Department of Medical Social Sciences, Northwestern University, Chicago, IL, USA; c Department of Family Medicine, Geffen School of Medicine, University of California, Los Angeles, Los Angeles, CA, USA; d Department of Dietetics and Nutrition, Robert Stempel College of Public Health, Florida International University, Miami, FL, USA; e Department of Epidemiology, Bloomberg School of Public Health, Johns Hopkins University, Baltimore, MD, USA; f Department of Epidemiology and Public Health, School of Medicine, University of Maryland, Baltimore, MD, USA; g Department of Medicine, Johns Hopkins School of Medicine, Baltimore, MD, USA; h Department of Medicine, University of British Columbia, Vancouver, Canada; i Department of Pediatrics, Keck School of Medicine, University of Southern California, Los Angeles, CA, USA; j Faculty of Health Sciences, Simon Fraser University, Burnaby, Canada; k School of Public Policy, Simon Fraser University, Vancouver, Canada; l Frontier Science Foundation, Amherst, NY, USA

**Keywords:** COVID-19, Vaccine hesitancy, Substance Use

## Abstract

**Objective:**

We describe the prevalence of COVID-19 vaccine uptake, substance use, and other factors associated with vaccine hesitancy among participants from nine North American cohort studies following a diverse group of individuals at risk for or living with HIV.

**Methods:**

Between May 2021 and January 2022, participants completed a survey related to COVID-19 vaccination. Participants included those with and without substance use. Those responding as ‘no’ or ‘undecided’ to the question “Do you plan on getting the COVID-19 vaccine?” were categorized as vaccine hesitant. Differences between groups were evaluated using chi-square methods and multivariable log-binomial models were used to calculate prevalence ratios (PR) of COVID-19 vaccine hesitancy with separate models for each substance.

**Results:**

Among 1,696 participants, COVID-19 vaccination was deferred or declined by 16%. Vaccine hesitant participants were younger, with a greater proportion unstably housed (14.8% vs. 10.0%; p = 0.02), and not living with HIV (48.% vs. 36.6%; p <.01). Vaccine hesitant participants were also more likely to report cannabis (50.0% vs. 42.4%; p = 0.03), methamphetamine (14.0% vs. 8.2%; p <.01), or fentanyl use (5.5% vs. 2.8%; p = 0.03). Based on multivariable analyses methamphetamine or fentanyl use remained associated with COVID-19 vaccine hesitancy (Adjusted PR = 1.4; 95% CI 1.1–1.9 and Adjusted PR = 1.6; 95% CI 1.0–2.6, respectively).

**Conclusion:**

As new COVID-19 vaccines and booster schedules become necessary, people who use drugs (PWUD) may remain vaccine hesitant. Strategies to engage hesitant populations such as PWUD will need to be tailored to include special types of outreach such as integration with substance use programs such as safe injection sites or recovery programs.

## Highlights

• COVID-19 vaccine uptake was significantly lower among people who use drugs (PWUD), especially those reporting methamphetamine and fentanyl use.
• Besides substance use, lower vaccine uptake was associated with younger age and belief in vaccine misinformation.
• As new COVID-19 vaccines become necessary strategies to engage PWUD will be necessary.

## 1 Introduction

Since SARS-CoV-2 and the ensuing COVID-19 pandemic were recognized in early 2020, nearly 100 million cases and more than one million deaths attributed to COVID-19 have been reported in the United States (US) as of August 2022 (Centers for Disease Control and Prevention, 2022). Vaccines against COVID-19 first became available in the US in December 2020 after the Food and Drug Administration granted emergency use authorization for the Pfizer–BioNTech and Moderna vaccines, with mass vaccination campaigns beginning within days (Baden et al., 2021, Polack et al., 2020, Sadoff et al., 2021). Initial vaccine eligibility was targeted based on COVID-19 risk status, however, by May 2021 vaccine eligibility was expanded to include those 12 years of age or older and as of June 2022 vaccine eligibility includes children 6-months and older (Centers for Disease Control and Prevention, 2022).

COVID-19 skepticism and SARS-CoV-2 vaccine hesitancy defined as “delay in acceptance or refusal of vaccination despite availability of vaccination services” are an ongoing impediment to the public health response to the pandemic (World Health Organization, 2014). Determinants of COVID-19 vaccine uptake have been mixed and dynamic, depending on timing, place, and context. Shortly after approval of the vaccine the Kaiser Family Foundation conducted a national poll, which showed that 62% of respondents were unsure of COVID-19 vaccine safety and efficacy, believing that socio-political pressures led to a rushed approval (Kaiser Family Foundation, 2020). Since, levels of vaccine hesitancy have declined and as of October 2022 the CDC reports that 78% of people over the age of 18 have completed the primary series of vaccination, with nearly 50% having been boosted (Centers for Disease Control and Prevention, 2022). However, an estimated 10% of adults in the US report that they will not get vaccinated, a figure which has held steady since late 2021 (Centers for Disease Control and Prevention, 2022). Factors found to be consistently associated with COVID-19 vaccine hesitancy include younger age, female sex, identifying as Black/African American or Hispanic/Latinx, lower perceived severity of COVID-19, concerns about vaccine side effects or harms, and not having any chronic medical conditions (King et al., 2021, Kociolek et al., 2021, Kreps et al., 2021, Tram et al., 2022, Wang and Liu, 2022).

People with HIV (PWH) as well as people who use drugs (PWUD) face greater risk of severe and complicated COVID-19 and thus represent priority groups for vaccination (Baillargeon et al., 2021, Danwang et al., 2022, Pavarin et al., 2022, Sörberg Wallin et al., 2022, Ssentongo et al., 2021). The relationship between HIV and substance use is myriad and complicated. Substance and injection drug use are also prevalent among PWH, with striking implications on HIV comorbidities through pathways of immunoregulation and inflammation (Cook et al., 2019, Friedman et al., 2003). Furthermore, PWUD not living with HIV might be immunocompromised or otherwise at increased risk for respiratory infections due to a number of mechanisms including smoking or snorting of drugs (Radke et al., 2014, Vasylyeva et al., 2020). However, data on the prevalence of vaccination among these groups are limited. In a survey conducted in June 2021, Menza and colleagues found that 62% of PWH in Oregon had received at least one dose of the COVID-19 vaccine, with lower odds of vaccination among those who reported injection drug use (Menza et al., 2022). In another survey among people who used injection drugs, vaccine uptake as of June 2021 was estimated at 68%, with COVID-19 attitudes and vaccine knowledge being the most important predictors of vaccine uptake (Cepeda et al., 2022). As new SARS-CoV-2 variants emerge and new vaccines become available, understanding COVID-19 vaccine hesitancy, especially among populations most vulnerable to negative outcomes related to SARS-CoV-2 infections remains critical.

The objective of this study was to describe the prevalence of COVID-19 vaccine uptake as well as factors associated with vaccine hesitancy among participants in nine cohort studies of HIV/AIDS in the context of substance use. Unique to our study is inclusion of vaccine uptake from a broad and diverse group of participants living with and without HIV as well as those with and without substance use, with detailed information on type of substances used.

## 2 Methods

### 2.1 Study setting and population

The Collaborating Consortium of Cohorts Producing NIDA Opportunities (C3PNO) was established in 2017 by the National Institute on Drug Abuse (NIDA) to enhance data sharing opportunities and facilitate collaborative research efforts among NIDA-supported cohorts that examine HIV/AIDS in the context of substance use. Details of the participating cohorts and other methodology have been previously described (Gorbach et al., 2021), but in summary, the consortium is comprised of nine NIDA cohorts located throughout North America (Baltimore, Chicago, Los Angeles, Miami, and Vancouver) with data linking across a wide range of behavioral, clinical, and biological data from individuals at high-risk for HIV or living with HIV. Some cohorts exclusively focus on injection drug users or those who are living with HIV, however, all cohorts include both substance using and non-substance using populations.

Starting in May 2020, C3PNO launched a COVID-19 survey in order to examine COVID-19 risk with each participating cohort administering the survey to a minimum of 200 participants, who were eligible if they: (1) were enrolled in one of the participating C3PNO cohorts; (2) had a study visit in the preceding 12 months prior to launch of the COVID survey; (3) were willing and able to complete the survey remotely. Starting in May 2021, questions specific to COVID-19 vaccination were added to the survey and this analysis includes the 1,696 unique participants who completed the survey between May 2021 and January 2022.

### 2.2 Study procedures and data collection

Participants provided written informed consent and the study was approved by the Institutional Review Board at each of the participating cohorts within the consortium. Questionnaires were administered remotely using an internet-based survey which was self-administered for participants that had computer and internet access (88%) or interviewer administered by telephone for participants with barriers to self-administration and internet access (12%). The survey took approximately 20 minutes to complete and participants were remunerated between $15 and $25 (depending on cohort) for their time. In addition to sociodemographic information, specific domains collected as part of the survey included current HIV status, substance use, mental health, COVID-19 experiences, and the pandemic impact on day-to-day life. COVID-19 specific measures included questions related to COVID-19 positivity, vaccine status, as well as vaccine beliefs. Vaccine status was based on the question “Do you plan on getting the COVID-19 vaccine?” with answer choices including ‘I have already received the vaccine’, ‘yes’, ‘no’, or ‘undecided.’ Those responding as ‘no’ or ‘undecided’ were categorized as vaccine hesitant. Additionally, vaccine hesitant participants were asked about reasons for hesitancy including concerns about safety, efficacy, side effects, and general lack of concern regarding COVID-19. Three items were used to assess belief in COVID-19 vaccine misinformation including belief that the vaccine contains a live virus, the vaccine causes infertility, and belief that there are out-of-pocket costs associated with receiving the vaccine. Participants also reported on substance use in the past month, with questions specific to the following substances: (1) cannabis; (2) methamphetamine; (3) heroin; (4) fentanyl; and (5) non-medical use of prescription opioids. Given our interest in assessing the association between regular substance use and vaccine hesitancy, those reporting daily or weekly use of a given substance were categorized as people engaging in substance use while those who reported using a given substance only once or never were categorized as non-users.

### 2.3 Statistical analysis

Univariate analyses provided descriptive statistics for the sample overall and by COVID-19 vaccination status. Comparisons of sociodemographic characteristics and substance use by COVID-19 status were based on t-tests, chi-square methods, and other non-parametric tests as appropriate. Stratified analysis allowed us to examine the potential differential impact of HIV status and substance use on COVID-19 vaccine hesitancy. The association between factors of interest and COVID-19 vaccine hesitancy was examined using log-binomial regression analyses. Separate multivariable models were developed for each substance in order to avoid collinearity related to the co-use of various substances. Univariate analyses along with *a priori* knowledge informed variables for inclusion in multivariable models. All analyses were conducted using SAS version 9.4 (SAS Inc., Cary, NC).

## 3 Results

### 3.1 Characteristics of the study population

Among the 1,696 participants who completed the survey, the median age was 47 years [interquartile range (IQR) 28–59], 57% identified as Black, non-Hispanic, followed by 19% White, non-Hispanic, and 19% Hispanic/Latinx (Table 1). Unstable housing defined as living in a shelter, transitional housing, street, vehicle, abandoned building, or group home was reported by 11% of participants. People living with HIV comprised nearly half of the respondents (46%) and recent substance use was common. Cannabis use in the past month was reported by 44% of participants, with 12% reporting cocaine use, 9% reporting methamphetamine use, and 5% reporting heroin use.

**Table 1:** Characteristics of C3PNO participants overall and by COVID-19 vaccine hesitancy status, May 2021 - January 2022.

			COVID-19 Vaccine Status						
	Total (n = 1,696*)		Vaccine Hesitant (n = 257*)		Received or Planning to Receive Vaccine (n = 1,403*)				
	n	%		n	%		n	%	p value
Age, years (median, IQR)	47 (28–59)		35 (27–54)		50 (28–59)	<0.01			
Female sex at birth	415	24.6		72	28.2		343	24.6	0.21
Race/Ethnicity									0.20
Black, non-Hispanic	645	56.5		155	61.0		777	56.2	
Hispanic/Latinx	316	18.9		37	14.6		263	19.0	
Other	86	5.2		16	19.5		66	4.8	
White, non-Hispanic	324	19.4		46	18.1		276	20.0	
Unstable Housing^	188	11.1		38	14.8		140	10.0	0.02
Unemployed	952	56.7		141	55.7		800	57.4	0.63
HIV-positive	777	46.0		94	36.6		677	48.3	<0.01
Substance use, past month									
Smoke (including e-cigs)	754	45.0		117	45.5		633	45.0	0.91
Cannabis	721	43.6		125	50.0		591	42.4	0.03
Methamphetamine	155	9.2		36	14.0		115	8.2	<0.01
Cocaine	195	11.6		33	12.8		160	11.4	0.51
Heroin	85	5.1		17	6.6		67	4.8	0.22
Fentanyl	54	3.2		14	5.5		39	2.8	0.03
Rx Opioids	80	4.8		15	5.8		64	4.6	0.38
Received Influenza Vaccine, past 5-years						<0.01			
Never	376	23.3		149	60.3		222	16.3	
Sometimes	231	14.3		37	15.0		194	14.2	
Most years	202	12.5		19	7.7		181	13.3	
Every year	807	49.9		42	17.0		765	56.2	
Belief in COVID-19 Vaccine Misinformation									
Vaccine contains live virus	183	11.4		57	24.5		121	8.9	<0.01
Vaccine causes infertility	70	4.3		35	15.0		31	2.3	<0.01
Vaccine has out-of-pocket cost	61	3.8		16	6.9		41	3.0	<0.01
Any of the above	230	14.4		66	28.2		159	11.7	<0.01

Abbreviations. IQR = Interquartile range.

*Sum may not equal due to missing data.

^unstable housing defined as living in shelter, transitional housing, street, vehicle, abandoned building, or group home.

### 3.2 COVID-19 vaccine hesitancy

Nearly 80% of participants reported being vaccinated for COVID-19. Vaccine hesitant participants were younger (median age 35 years vs. 50 years; p <.01), with no differences noted by sex or race/ethnicity (Table 1). Further, vaccine hesitancy was less common among PWH (p <.01). COVID-19 vaccine hesitancy was also correlated with receipt of the influenza vaccine. For instance, 60% of those who were hesitant to receive the COVID-19 vaccine reported never receiving the influenza vaccine in the past five years as compared to 16% among those who received the COVID-19 vaccine (p <.01).

**Fig. 1:**
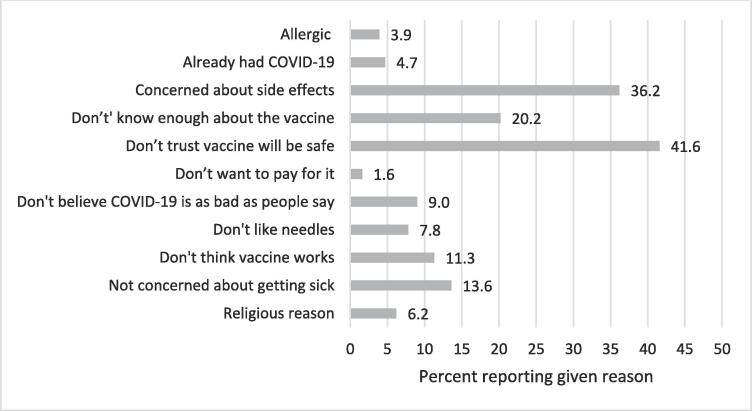
Reasons for declining or deferring COVID-19 vaccination among C3PNO participants, May 2021-January 2022.

Among the 16% (n = 257) who declined vaccination, the most common reasons centered around side effects and safety, with 42% reporting that they “don’t trust the vaccine will be safe,” 36% reporting “concerns about side effects,” and 20% reporting that they did not feel informed enough about the vaccine (Fig. 1). Endorsement of statements regarding vaccine misinformation were reported by 14% (n = 230) of participants, among whom the most prevalent belief was that the vaccine contained a live virus (80%), followed by the belief that the vaccine would cause infertility (31%).

**Fig. 2:**
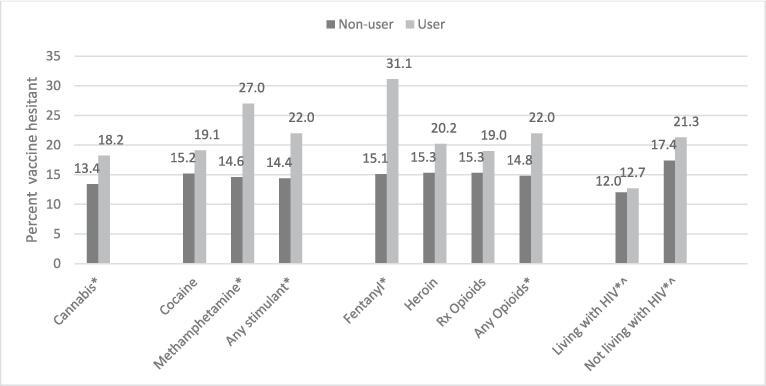
Prevalence of COVID-19 vaccine hesitancy by substance use status among C3PNO participants, May 2021-January 2022.

Higher prevalence of vaccine hesitancy was noted among those who reported substance use. Nearly one in four participants who reported methamphetamine use reported refusing or being undecided about the COVID-19 vaccine as compared to 15% of participants who did not report methamphetamine use (p <.01)(Fig. 2). A similar trend was noted for those who reported opioid use, including those who reported fentanyl, heroin, or prescription opioid misuse. Analyses stratified by HIV status show that vaccine hesitancy was lowest among PWH, particularly those who reported no substance use (12%) increasing to 21% among participants not living with HIV but who reported substance use (Fig. 2).

Based on multivariable analyses adjusting for HIV-status, sociodemographic factors independently associated with COVID-19 vaccine hesitancy included age and sex (Table 2). In particular we found that the probability of vaccine hesitancy was higher among those in the younger age groups and females had 1.5 times the likelihood of vaccine hesitancy as compared to males [adjusted prevalence ratio (APR) = 1.5; 95% confidence interval (CI) 1.2–2.0). Separate multivariable models for each specific type of substance shows that after adjusting for age, sex, and HIV-status, those who reported methamphetamine use were more likely to be vaccine hesitant (APR = 1.4; 1.1–1.9) as compared to those who did not report methamphetamine use. Likewise, those who reported fentanyl use had were nearly twice as likely to report vaccine hesitancy as compared to those who did not report fentanyl use (APR = 1.6; 95% CI 1.0–2.6).

**Table 2:** Multivariable log-binomial regression analysis of factors associated with COVID-19 vaccine hesitancy among C3PNO participants.

												
	**Adjusted PR (95% CI)**			**Adjusted PR (95% CI)**			**Adjusted PR (95% CI)**			**Adjusted PR (95% CI)**			**Adjusted PR (95% CI)**			**Adjusted PR (95% CI)**		
Sex at birth																		
Female	1.5	(1.2–2.0)		1.5	(1.2–2.0)		1.5	(1.1–2.0)		1.5	(1.2–1.9)		1.5	(1.1–1.9)		1.5	(1.1–1.9)	
Male	1.0	Reference		1.0	Reference		1.0	Reference		1.0	Reference		1.0	Reference		1.0	Reference	
																		
Age																		
18–29 years	1.5	(1.1–2.1)		1.6	(1.2–2.2)		1.6	(1.2–2.3)		1.6	(1.2–2.3)		1.6	(1.2–2.3)		1.6	(1.2–2.3)	
30–49 years	1.9	(1.4–2.5)		1.9	(1.4–2.5)		2.0	(1.5–2.6)		2.0	(1.5–2.6)		1.9	(1.4–2.6)		2.0	(1.5–2.6)	
>50 years	1.0	Reference		1.0	Reference		1.0	Reference		1.0	Reference		1.0	Reference		1.0	Reference	
																		
HIV status																		
HIV-negative	1.4	(1.1–1.8)		1.4	(1.1–1.8)		1.4	(1.1–1.8)		1.4	(1.1–1.8)		1.4	(1.1–1.8)		1.4	(1.1–1.8)	
HIV-positive	1.0	Reference		1.0	Reference		1.0	Reference		1.0	Reference		1.0	Reference		1.0	Reference	
																		
Substance Use*																		
Cannabis	1.2	(0.9–1.5)		–	–		–	–		–	–		–	–		–	–	
Methamphetamine	–	–		1.4	(1.1–1.9)		–	–		–	–		–	–		–	–	
Cocaine	–	–		–	–		1.2	(0.8–1.5)		–	–		–	–		–	–	
Heroin	–	–		–	–		–	–		1.1	(0.7–1.7)		–	–		–	–	
Fentanyl	–	–		–	–		–	–		–	–		1.6	(1.0–2.4)		–	–	
Rx Opioids	–	–		–	–		–	–		–	–		–	–		1.2	(0.7–1.9)	

*Abbreviations. PR = Prevalence ratio; CI = Confidence Interval.

*Separate models were developed for each substance and PR adjust for sex, age, and HIV-status.

## 4 Discussion

Findings from this study show that 16% of participants interviewed between May 2021 and January 2022 were not vaccinated against COVID-19 and did not plan on receiving the vaccine. While seemingly lower when compared to earlier studies, vaccine hesitancy among our study population remains higher than the 10% noted among the US general population (Centers for Disease Control and Prevention, 2022, Cepeda et al., 2022, Menza et al., 2022). Additionally, we demonstrate that a number of factors were independently associated with COVID-19 vaccine hesitancy including younger age, not living with HIV, and current substance use, especially methamphetamine and fentanyl use. Other studies have noted an association between injection drug use and low COVID-19 vaccine uptake, however, this is one of the first to further explore specific types of substances used and show that stimulant use including methamphetamine use is associated with lower vaccine uptake (Cepeda et al., 2022, Strathdee et al., 2021).

Our finding that PWH were more likely to be vaccinated is in line with other studies, which note that COVID-19 vaccine uptake is higher among people living with co-morbid conditions (including HIV)(Wang and Liu, 2022, Wickersham et al., 2022). However, PWUD, another group especially vulnerable to the negative outcomes associated with COVID-19 had significantly lower levels of vaccine uptake especially those reporting methamphetamine and fentanyl use. Stigma and marginalization are not uncommon among PWUD, who may in turn be less willing to follow public health directives, especially if the government is not viewed as a collaborator or steward of public health and safety. Often existing beyond the reach of public health systems and care networks, this marginalization or alienation has especially been exacerbated by the global shutdown and social and emotional distancing mandated with the COVID-19 pandemic. The fact that nearly 80% of PWUD received vaccination is a testament to the concerted public health vaccination efforts. However, going forward strategies that account for specific barriers of vaccine uptake among this population are needed, including strategies that address structural barriers and potential mistrust of the medical and public health system (Barocas, 2021). One potential approach might involve combining trusted sources such as sponsors and recovery coaches to serve as vaccine champions, while locations that house these trusted relationships such as substance use treatment centers can serve as vaccination delivery sites. The potential utility of this approach is supported by evidence indicating that delivery of vaccines in non-traditional settings has been successful for other vaccinations including influenza and hepatitis B (Coady et al., 2007, Des Jarlais et al., 2001). While this approach may be useful in reaching those who are in recovery or receiving treatment for substance use, the use of peer ambassadors and other trusted messengers could be key to delivering services to PWUD not in care. A study of peer ambassadors promoting COVID-19 vaccine among those experiencing homelessness found that the pre-existing trust and shared experiences of homelessness among peer ambassadors not only made the program successful, it helped foster opportunities to make the vaccine advocacy program more sustainable and equitable (Choi et al., 2022).

Our findings that prior influenza vaccination was associated with COVID-19 vaccine uptake, while belief in vaccine misinformation was associated with COVID-19 vaccine hesitancy, have been noted in other studies (Loomba et al., 2021, Wickersham et al., 2022). However, it is likely that these associations underlie the confluence of a number of other factors, particularly those related to social determinants of health. For instance, people vaccinated against influenza may also represent groups with higher levels of access to care and care engagement. Indeed, this was the case with our study population with those consistently vaccinated against influenza in the past five years, more likely to report having a doctor’s visit in the past year (data not shown). In addition to access issues, people not vaccinated represent a group that has less confidence in the safety and effectiveness of the vaccine (Aw et al., 2021, Wang and Liu, 2022). Safety concerns were top among reasons noted among our participants, but other misinformation such as infertility and out of pocket costs were also noted suggesting that an understanding of the specific reason for hesitancy will help delineate belief structures that serve as barriers to vaccine uptake and help target interventions. Not only does exposure to misinformation result in a decrease in vaccine acceptance, but this decline occurs regardless of pre-exposure intent (Loomba et al., 2021). Furthermore, there is a differential impact of belief in misinformation with those who report trust in non-expert sources such as family and friends or overconfidence in one’s knowledge base being more susceptible to belief in COVID-19 vaccine misinformation (Loomba et al., 2021, Motta et al., 2018). Taken together, this suggests that using sources of trusted information can not only help increase knowledge but also reduce the potential impact of vaccine misinformation.

This study had several limitations. First, the data for this study are based on self-report and participants may under report socially stigmatized behaviors or attitudes such as substance use and vaccine hesitancy. However, the use of computer-assisted self-interviews (with the majority of respondents) may help minimize the potential for social desirability bias. Second, vaccine hesitancy is a dynamic construct and given the cross-sectional nature of our study, it is possible that some participants who expressed a reluctance to receive the COVID-19 vaccine as part of our study, could have been subsequently vaccinated. Finally, a convenience sample of participants from each cohort completed this survey and those included in this study may not be representative of the parent cohorts. Additionally, participants from each cohort may not be generalizable to populations living with HIV or other substance-using populations in North America. Nonetheless, the study is strengthened by data on specific substances used among a relatively large sample size with inclusion of a socio-demographically diverse group of people living at the intersection of multiple vulnerabilities of substance use and HIV.

## 5 Conclusion

In conclusion, this study demonstrates that as vaccination efforts for COVID-19 continue, especially as new vaccines and booster schedules become necessary, a substantial proportion of a vulnerable population remains vaccine reluctant. Given the large number of people affected by substance use, public health measures to improve vaccine coverage, especially among those not actively in care (such as PWH) are needed. Tailored approaches such as integration into substance use treatment and proactive outreach from trusted sources are needed to address the complex phenomenon of vaccine hesitancy among an especially vulnerable population.

## Funding sources

This work was supported by the National Institute on Drug Abuse (NIDA) of the following National Institutes of Health under award numbers: U24DA044554; U01DA036926; U01DA036935; U01DA040381; U01DA036267; U01DA036939; U01DA036297; U01DA021525; U01DA040325; U01DA038886.

## Declaration of Competing Interest

The authors declare that they have no known competing financial interests or personal relationships that could have appeared to influence the work reported in this paper.

## Data Availability

Data will be made available on request.
